# Evaluation of Dual Time Point Imaging 18F-FDG PET/CT in Differentiating Malignancy From Benign Gastric Disease

**DOI:** 10.1097/MD.0000000000001356

**Published:** 2015-08-21

**Authors:** Jing Cui, Panxiong Zhao, Zhentai Ren, Baoping Liu

**Affiliations:** From the Department of Nuclear Medicine, The First Affiliated Hospital of Zhengzhou University, Henan, China (JC, BL); Department of Nuclear Medicine, The First Affiliated Hospital of Henan University, Henan, China (PZ); and Department of Radiation Oncology, The Fifth Affiliated Hospital of Zhengzhou University, Henan, China (ZR)

## Abstract

To assess the clinical value of dual time point imaging (DTPI) fluorine-18fludeoxyglucose (18F-FDG) positron emission tomography (PET)/CT in differentiating malignancy and benign disease of patients with focally increased gastric uptake.

Patients who present focally increased 18F-FDG uptake in gastric wall on conventional PET/CT imaging received delayed imaging. PET/CT scans were acquired at 1 and 2 hours (early and delayed imaging) after 18F-FDG injection. The maximum standardized uptake value (SUV) was calculated. The SUVmax of the early and delayed imaging acquisition were signed S1 and S2, respectively. The receiver operating characteristic curve (ROC) of the S1, S2, and the retention index (RI) were drawn to find the best cut-off point value for differential diagnosis. Sensitivity, specificity, Youden index, and the area under the curve (AUC) were calculated, respectively.

From September 2010 to May 2015, 74 patients (56 male and 18 female; age of 57 ± 12 years; range, 32–86 years) referring for areas of focally increased uptake of 18F-FDG in gastric wall received delayed imaging. The S1 was 5.0 ± 1.4 (range, 1.9–11.3), and S2 was 5.9 ± 2.7 (range, 1.0–16.3). The SUVmax were increased in 52 patients in delayed imaging, with 85% (44/52 cases) appeared malignant; decreased in 20 patients, and 90% (18/20 cases) were benign; 2 patients of benign had not changed. The change of SUVmax between malignant and benign was significant difference (*t* = −5.785, *P* = 0.000).Taking the S1, S2, and RI higher than 4.6%, 5.1%, and 13% as positive diagnostic criteria, the sensitivity were 65.2%,87.0%, and 87.0%, respectively; the specificity were 64.3%, 82.1%, and 89.3%; the Youden index were 0.332, 0.693, and 0.770; AUC were 0.635 (95% confidence intervals (95% CI) 0.507–0.764), 0.873 (95% CI, 0.786–0.961), and 0.923 (95% CI, 0.854–0.992).

DTPI is more precise to distinct malignant from benign gastric diseases compared with conventional imaging, and it is readily accessible.

## INTRODUCTION

Gastric cancer, the fourth leading cause of cancer-related mortality worldwide, continues to be an important healthcare problem from a global perspective, though its incidence and mortality decreased substantially over the last decades in most countries worldwide.^1,2^ Early detection and surgery can extremely improved the results of treatment, therefore, improve diagnosis is one of the most important way to further reduce the burden of gastric cancer.^1^ Conventional imaging of gastric cancer with fluorine-18fludeoxyglucose (18F-FDG) positron emission tomography (PET) is not successful because of the limited sensitivity and accuracy, and the FDG uptake is strongly related to some factors, such as tumor size, location, histopathology.^3,4^

Some studies tried to explore novel approaches, such as gastric distention, it cannot significantly improve the diagnostic accuracy, though display the lesions more clearly^5,6^; 3-dimensional CT gastrography^7^ is the more intuitive and comprehensive method than conventional PET/CT in the diagnosis of gastric cancer, but it is only an improvement of using CT 3D technology, and few benign disease have local thickening gastric wall. In recent years, multiple studies have shown the dual time point imaging (DTPI) of FDG PET may be helpful in differentiating malignancy from benign processes, thus enhancing the diagnostic accuracy of FDG PET,^8–11^ but few focus on gastrointestinal tract.^12^ No previous studies have addressed the use of DTPI for gastric disease. The purpose of this study was to investigate the usefulness of DTPI in patients with suspected gastric cancer, and to assess the clinical value of the delayed imaging compared with the early ones.

## MATERIALS AND METHODS

This retrospective study was exempt from approval by the hospital review boards of the First Affiliated Hospital of Zhengzhou University, and no informed consent was needed. The research procedures followed were in accordance with the Helsinki Declaration.

### Patients

From September 2010 to May 2015, 91 patients who referring for areas of focally increased 18F-FDG uptake in gastric wall that exceeding the surrounding normal tissue received delayed imaging. Seventeen patients with a history of other types of cancers were excluded. Therefore, 74 patients were eligible in this retrospective study. All these 74 patients (56 male and 18 female; age of 57 ± 12 years; range, 32–86 years) had pathology or confirmed by endoscope and clinical follow-up.

### DTPI PET/CT Techniques

All patients fasted for at least 6 hours before PET/CT scans, and blood glucose levels were less than 150 mg/dL before injection of 18F-FDG (the radiochemical purity > 95%). Whole body static emission scans were performed twice (at 1 and 2 hours)^13^ after intravenous injection of 3.7 MBq/kg 18F-FDG. Just before imaging, patient was required to drink 500 mL water for fulfill stomach. Imaging was obtained by Biography TruePoint 64 PET/CT (Siemens, Germany). All PET/CT scans were performed in 3-dimensional mode with a matrix of 128 × 128 and an acquisition time of 2.0 minutes/bed position. Low-dose CT parameters for body-scan were 120 kV, 0.8 s tube rotation, 3.0 mm section thickness, and electric current depending on anatomic location using an automated exposure control system. One hour later, reposition patients to obtain the delayed imaging by using the same parameters. Semi-quantitative analysis involved mainly transverse and coronal images with the Syngo workstations.

### PET Data Analysis

All 18F-FDG PET imaging were evaluated by 2 experienced nuclear medicine specialists who were unaware of the clinical data and the results of other imaging studies. Positive FDG uptake was defined as the thickened gastric wall was greater than that of the adjacent gastric wall. The maximum standardized uptake value (SUV) was calculated using the following formula^14^: SUV = c_dc_/(d_i_/w), where c_dc_ is the decay-corrected tracer tissue concentration (in Bq/g), d_i_ is the injected dose (in Bq), and w is the patient's body weight (in g). The retention index (RI) was calculated by subtracting the SUVmax1 from the SUVmax2 and dividing by SUVmax1. For lesions visible on both or either of the 2 PET/CT scans, an ROI was drawn on the respective imaging in the region corresponding to the area of abnormality on the PET/CT imaging.

### Statistical Analysis

Statistical analyses were performed using SPSS version 17.0 software. Quantitative values were expressed as mean ± SD. Comparison between groups was performed using independent samples *t* test and 1-way ANOVA. The SUVmax for the diagnosis of gastric cancer was analyzed by a receiver operating characteristic curve (ROC). All analyses were 2-sided, a *P*-value of less than 0.05 was considered statistically significant.

## RESULTS

**FIGURE 1:**
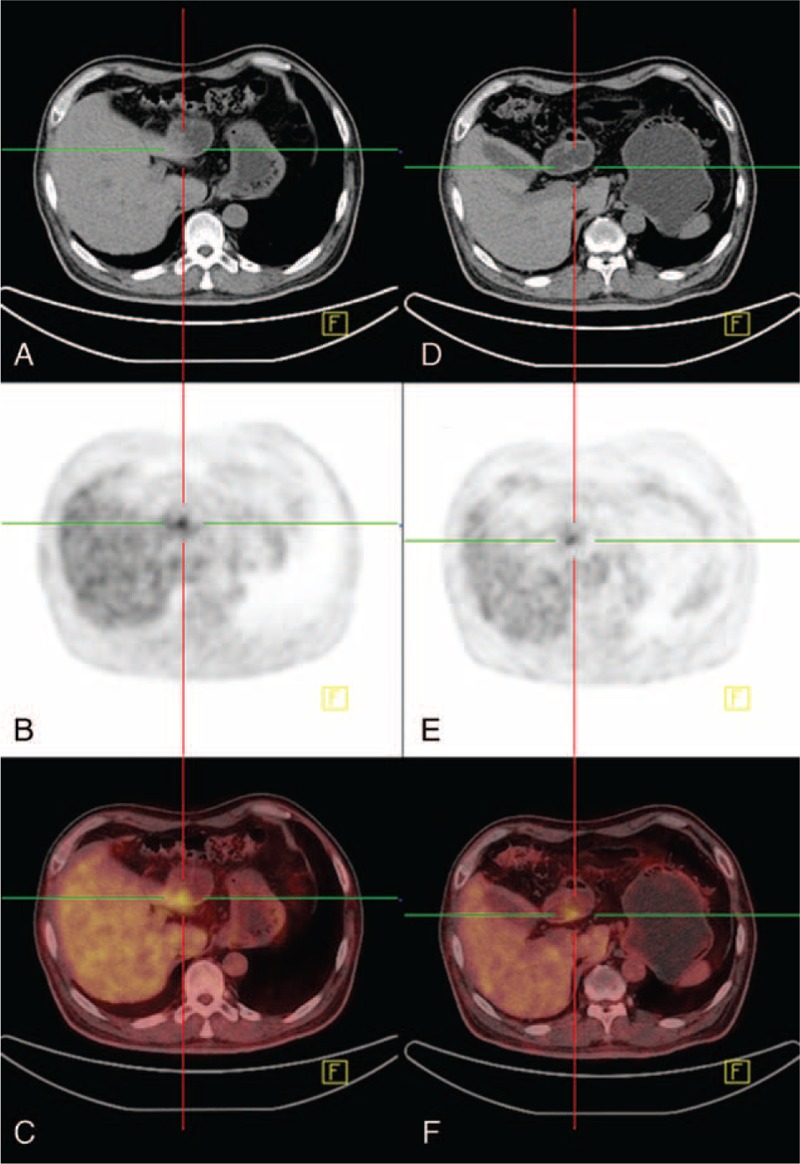
A 58-year-old man was found focally increased 18F-FDG uptake in gastric antrum on PET/CT health check, S1 was 5.2 (A, CT image; B, PET image; C, PET/CT fusion image) on conventional imaging at 60 minutes after tracer injection. Delayed imaging (D, CT image; E, PET image; F, PET/CT fusion image) was performed 65 minutes later, and S2 was 4.7. Both scans showed gastric wall thickening in local gastric antrum. Gastric endoscopy suggested superficial gastritis.

**FIGURE 2:**
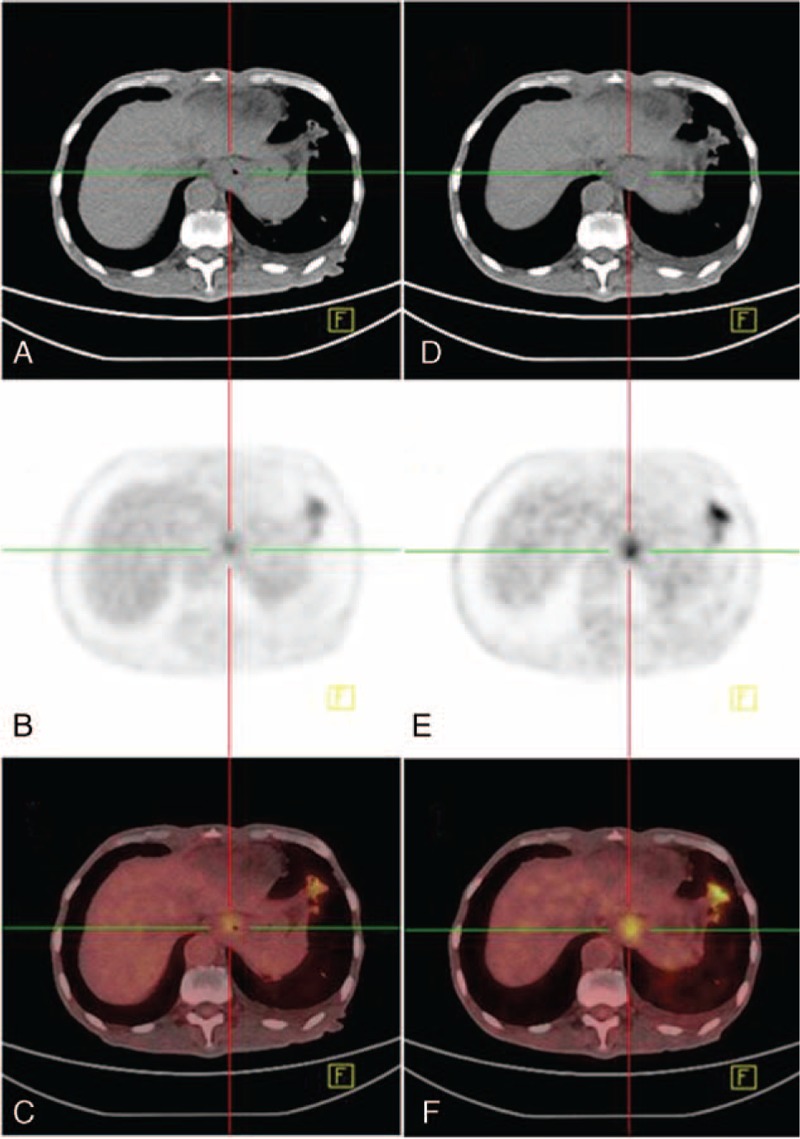
A 75-year-old man with progressively difficulty eating could not received endoscopy biopsy because of cardinal stricture. The early (A–C) and delayed (D–F) imaging were obtained 56 and 137 minutes after tracer injection, S1 was 4.2 and S2 was 5.5, respectively. A moderately differentiated tubular adenocarcinoma of the cardia was confirmed by surgery.

**FIGURE 3:**
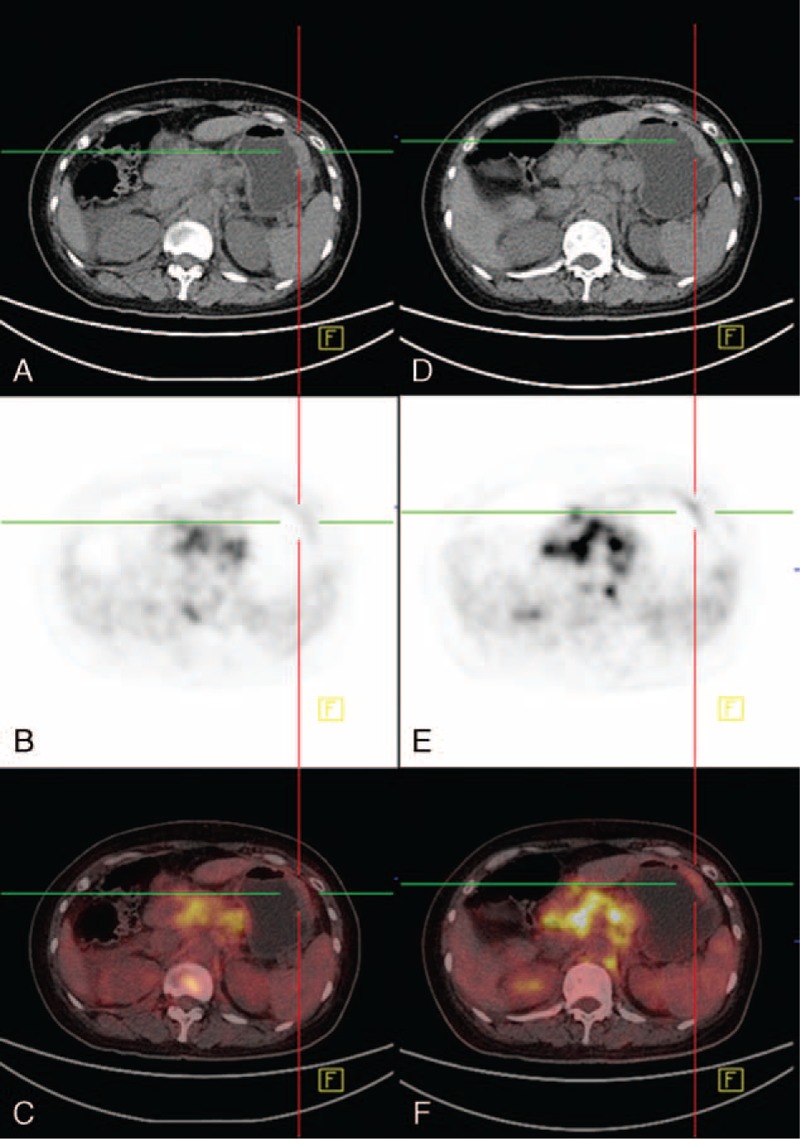
A 51-year-old woman with retroperitoneal lymph node enlargement received PET/CT to find suspicious primary tumors. The early (A–C) and delayed (D–F) imaging were obtained 64 and 142 minutes after tracer injection, SUVmax of diffuse thickening gastric wall was S1 1.9 and S2 3.8, respectively. A poorly differentiated tubular adenocarcinoma of the greater curvature side was confirmed by endoscopy biopsy.

In all 74 patients, 46 cases were proved to be malignant and 28 cases were benign, details of histopathology based on the WHO classification are listed in Table 1. The mean FDG uptake of S1 was 5.0 ± 1.4 (range, 1.9–11.3), and S2 was 5.9 ± 2.7 (range, 1.0–16.3). The change of SUVmax in delayed imaging was significantly (*t* = −3.339, *P* = 0.002). Early focal 18F-FDG uptake in the benign and malignant sites showed no significant difference (*t* = −1.780, *P* = 0.081), and in delayed imaging the difference was significantly (*t* = −4.651, *P* = 0.000). The change of SUVmax between benign (Figure 1) and malignant groups (Figures 2 and 3) was significantly (*t* = −5.785, *P* = 0.000). The SUVmax were increased in 52 patients in delayed imaging, with 85% (44/52 cases) appeared malignant; decreased in 20 patients, and 90% (18/20 cases) were benign; 2 patients of benign had not changed.

**Table 1:**
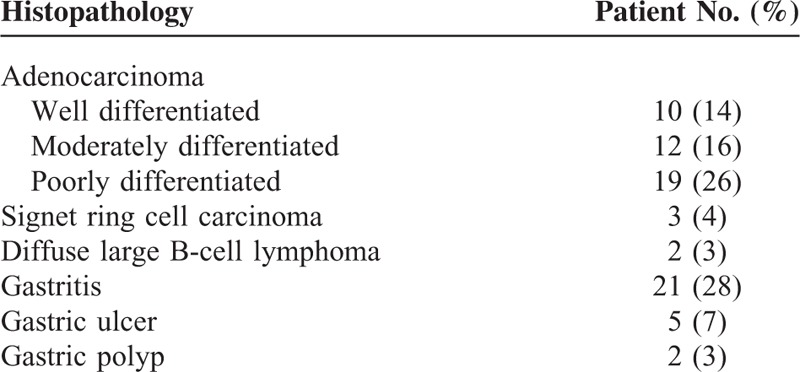
Patients Histopathology

The SUVmax of 2 diffuse large B-cell lymphoma lesion were increased to 7.1 and 12.8 at delayed imaging from 5.7 and 9.9 at early imaging. Only 1 malignant lesion in 3 with signet cell carcinoma had a slightly decrease (S1 5.7 vs. S2 5.6), while other 2 had increased (S1 3.2 vs. S2 4.4); (S14.9 vs. S2 5.6). In 8 benign foci with increased SUVmax at delayed imaging, 6 foci were gastritis; the other 2 was gastric ulcer. The SUVmax on early imaging of different adenocarcinoma types had no statistical significance (*F* = 2.917, *P* = 0.233), there were no significant difference among their RI (*F* = 2.097, *P* = 0.125) also.

**FIGURE 4:**
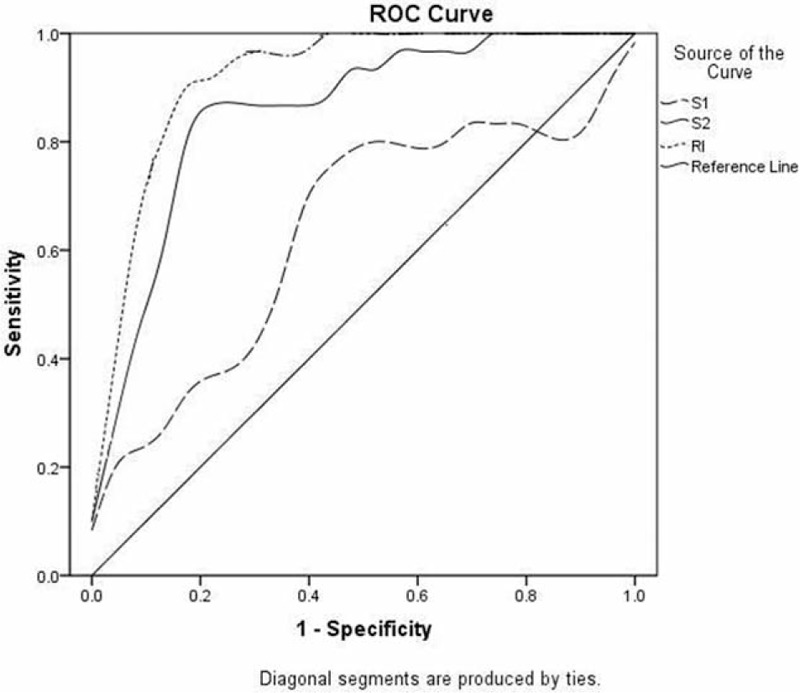
ROC curves for DTPI 18F-FDG PET/CT imaging.

The ROC curves for S1, S2, and RI of 18F-FDG PET/CT imaging are shown in Figure 4. The sensitivity, specificity, Youden index, and the area under the curve (AUC) of S1, S2, and RI are shown in Table 2. Taking the S1, S2, and RI higher than 4.6%, 5.1%, and 13% as positive diagnostic criteria, the sensitivity were 65.2%,87.0%, and 87.0%, respectively; the specificity were 64.3%, 82.1%, and89.3%; the Youden index was 0.332, 0.693, and 0.770; AUC were 0.635 (95% confidence intervals (95% CI), 0.507–0.764), 0.873 (95% CI, 0.786–0.961), and 0.923 (95% CI, 0.854–0.992).

**Table 2:**
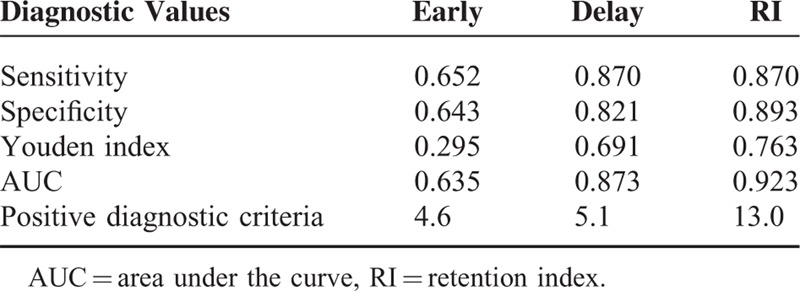
Diagnostic Values of Early and Delayed FDG Imaging of Gastric Disease

## DISCUSSION

Recently, detection and diagnosis of gastric cancer remains the domain of conventional imaging modalities such as endoscopy, endoscopic ultrasound, and CT.^15^ FDG-PET has rarely been used as a diagnostic modality for gastric cancer mainly due to limited resolution, sensitivity, and accuracy, and imaging is mainly used to stage the gastric cancer rather than screening, in addition, FDG was found to have extremely different accumulation in different gastric cancer.^4^ Published sensitivities for 18F-FDG PET range from 37.9% to 86.1%^3,7,15–18^ for the detection of gastric cancer, which make primary staging and early evaluation of response to treatment impossible. The consideration of relatively large range sensitivity were advanced PET/CT and image postprocessing technique (eg, hybrid PET/CT 3-dimensional CT gastrography^7^); new scanning technique (eg, gastric distention^5,6^); the staging of gastric cancer (early or advanced gastric cancer^7^); selection bias because of limited cases and retrospective analysis.

In this study, the sensitivity on early imaging was 65.2%, and AUC was only 0.635 (95% CI, 0.507–0.764), numerous benign cases might cause increased FDG uptake indistinguishable from that of malignancy. Therefore, it is hard to distinguish malignancy from benign disease with suspected 18F-FDG uptake on early imaging; and any SUVmax cut-off value on early imaging as a diagnostic criterion is unacceptable. For delayed imaging, the sensitivity and AUC had significant improved to 87.0% and 0.873 (95% CI, 0.786–0.961); and RI (>13%) could achieve 0.923 (95% CI, 0.854–0.992), therefore, delayed 18F-FDG PET/CT imaging might be considered when conventional imaging is indeterminate. RI >13% is probably the rational criterion as an indication of malignancy.

Multiple studies have indicated that lesions of acute inflammation and infection may have higher FDG activity on delayed imaging, similar to that in malignancy, that might due to the different inflammatory cells involved.^8^ The underlying rationale of DTPI is that the uptake and clearance depend on the time interval between intravenous FDG administration and imaging.^8^ Enhanced glycolysis is a unique characteristic of cancer cells^9^; on delayed imaging, tissues with high glycolysis may have continuously increasing amounts of FDG trapped in cells in the form of FDG-6-phosphate, while tissues with high glucose-6-phosphatase activity will have an early peak followed by a gradual decrease in intracellular FDG retention.^8^ Increased cell proliferation rate and enhanced expression of hexokinase type-II and glucose transporter-1 may also contribute to increased FDG uptake in tumor cells on delayed imaging.^19,20^ And longer time of imaging allows improved blood pool and urinary tract clearance, thus lower FDG back ground. Most normal tissues and benign diseases have decreased background activity and most malignant ones have increased FDG uptake on delayed imaging, leading a higher lesion-to-background ratios, thus higher sensitivity.^8^

Some previous studies reported signet ring cell carcinoma and poorly differentiated adenocarcinomas are non-FDG avid, and always had a lower FDG uptake.^21,22^ In this study, the S1 of 3 different adenocarcinomas have no difference with each other, the same with RI. Although the number of cases is 3, there also showed increased SUVmax in 2 patients with signet ring cell carcinoma.

There are few studies concern about 18F-FDG PET/CT DTPI in gastrointestinal tumors. In the use of colorectal cancer,^12^ delayed imaging yielded information useful for differentiating physiologic uptake from pathological uptake and reduced false-positives in the abdomen. This clinical study demonstrated the potential use of dual-phase 18F-FDG PET/CT for gastric cancer. In contrast to the early imaging, delayed was able to detect more locally gastric cancers with sufficient contrast for quantification. DTPI may helpful to avoid unnecessary invasive examinations.

As a retrospective study, there may have been a selection bias.

## CONCLUSION

DTPI is a useful technique in differentiating benign from malignant gastric cases. It has a higher sensitivity and accuracy than conventional imaging, and it is readily accessible.
